# Liposomal Nanoformulation as a Carrier for Curcumin and pEGCG—Study on Stability and Anticancer Potential

**DOI:** 10.3390/nano12081274

**Published:** 2022-04-08

**Authors:** Ludwika Piwowarczyk, Malgorzata Kucinska, Szymon Tomczak, Dariusz T. Mlynarczyk, Jaroslaw Piskorz, Tomasz Goslinski, Marek Murias, Anna Jelinska

**Affiliations:** 1 Chair and Department of Pharmaceutical Chemistry, Poznan University of Medical Sciences, Grunwaldzka 6, 60-780 Poznan, Poland; szymon.tomczak@ump.edu.pl (S.T.); ajelinsk@ump.edu.pl (A.J.); 2 Chair and Department of Toxicology, Poznan University of Medical Sciences, Dojazd 30, 60-631 Poznan, Poland; kucinska@ump.edu.pl (M.K.); marek.murias@ump.edu.pl (M.M.); 3 Chair and Department of Chemical Technology of Drugs, Poznan University of Medical Sciences, Grunwaldzka 6, 60-780 Poznan, Poland; mlynarczykd@ump.edu.pl (D.T.M.); tomasz.goslinski@ump.edu.pl (T.G.); 4 Chair and Department of Inorganic & Analytical Chemistry, Poznan University of Medical Sciences, Grunwaldzka 6, 60-780 Poznan, Poland; piskorzj@ump.edu.pl

**Keywords:** bladder cancer, curcumin, epigallocatechin gallate, liposomes, prostate cancer, stability

## Abstract

Nanoformulations are regarded as a promising tool to enable the efficient delivery of active pharmaceutical ingredients to the target site. One of the best-known and most studied nanoformulations are liposomes—spherical phospholipid bilayered nanocarriers resembling cell membranes. In order to assess the possible effect of a mixture of polyphenols on both the stability of the formulation and its biological activity, two compounds were embedded in the liposomes—(i) curcumin (CUR), (ii) a peracetylated derivative of (−)-epigallocatechin 3-*O*-gallate (pEGCG), and (iii) a combination of the aforementioned. The stability of the formulations was assessed in two different temperature ranges (4–8 and 20 °C) by monitoring both the particle size and their concentration. It was found that after 28 days of the experiment, the liposomes remained largely unchanged in terms of the particle size distribution, with the greatest change from 130 to 146 nm. The potential decomposition of the carried substances was evaluated using HPLC. The combined CUR and pEGCG was sensitive to temperature conditions; however its stability was greatly increased when compared to the solutions of the individual compounds alone—up to 9.67% of the initial concentration of pEGCG in liposomes after 28 days storage compared to complete decomposition within hours for the non-encapsulated sample. The potential of the prepared formulations was assessed in vitro on prostate (LNCaP) and bladder cancer (5637) cell lines, as well as on a non-cancerous human lung fibroblast cell line (MRC-5), with the highest activity of IC_50_ equal 15.33 ± 2.03 µM for the mixture of compounds towards the 5637 cell line.

## 1. Introduction

Cancers of the urogenital system include, among others, prostate cancer and bladder cancer (BC) [1]. The urinary bladder is a hollow organ of muscle tissue located in the lower abdomen behind the symphysis pubis. Bladder cancer is a heterogeneous disease and is a spectrum of lesions of varying degrees of malignancy, infiltration depth, and disease progression risk. BC is the tenth most common cancer globally, and its incidence continues to increase worldwide. The increased risk of developing bladder cancer is more common in heavy smokers and also in men than in women. Southern Europe is one of the regions with the highest incidence of bladder cancer, with 26.5/100 000 men and 5.5/100 000 women each year developing the disease. Other factors include exposure to carcinogens (e.g., aromatic amines), *Schistosoma haematobium* infection, past bladder irradiation, prolonged and recurrent cystitis, and long-term use of a bladder catheter [2,3,4].

There are non-muscle-invasive (NMIBC) and muscle-invasive tumors (MIBC) in BC. Patients with confirmed bladder cancer are classified by tumor grade and by tumor stage, according to the recently published American Joint Committee on Cancer (AJCC). Moreover, BC treatment is dependent on the tumor-node-metastasis(TNM) staging system (Ta, Tis, T1–T4) and other factors like the patient’s overall condition and age, and the tolerability of the treatment method [5,6]. The type of NMIBC is treated with transurethral resection, most commonly followed by immunotherapy using the Bacillus Calmette-Guérin (BCG) vaccine or intravesical chemotherapy. In contrast, MIBC is usually treated with radical cystectomy and neoadjuvant chemotherapy due to higher rates of progression and relapse [7]. The administration of standard chemotherapeutic drugs causes many side effects, while immunotherapy can cause local irritation of the bladder epithelium. Relapses of the disease are frequent and require repeated resections, and therefore, it is desirable to look for new treatment options to prevent such relapses and metastasis [8]. The prostate is a single muscle-glandular organ, which is part of the male reproductive system. Prostate cancer was among the most common cancers diagnosed in men in the United States in 2017. The therapeutic potential of prostate cancer has improved significantly in recent years, although, it is still necessary to search for new drugs, especially in patients with advanced forms of the disease [9].

Polyphenols constitute a large and diverse group of organic compounds characterized by hydroxyl groups attached to an aromatic ring. Various studies indicate that the consumption of polyphenols may play an essential role in the regulation of the metabolism, as well as chronic and neoplastic disease treatment. At present, more than 8 000 polyphenols have been identified, but their impact on human health is not yet fully understood [10]. (−)-Epigallocatechin-3-*O*-gallate (EGCG) is a polyphenol found in abundance in green tea (*Camellia sinensis*) leaves, which exhibits pleiotropic biological activity, including anti-inflammatory and anticancer effects [11]. The most significant obstacles to the widespread use of this compound are its low oral bioavailability and also its chemical instability, which is induced by two major processes, epimerization, and auto-oxidation (in phosphate-buffered saline—60 mM, pH 7.4—at 37 °C, the stability of EGCG is only 1.5 h) [12].

Curcumin (CUR) is a polyphenolic compound with characteristic yellow color, obtained by extracting turmeric rhizomes (*Curcuma longa*), and consisting of two feruloyl residues linked by a methylene group. It has antioxidant, anti-inflammatory, antibacterial, and antiviral properties, as well as having shown to have potent anti-cancer activity. It is a promising drug candidate in liver and kidney disease, diabetes, cardiovascular diseases, arthritis, psoriasis, and neurodegenerative diseases, such as Alzheimer’s. It is also noteworthy that it is safe in large doses up to 12 g/24 h. The main factors limiting the use of curcumin is its low bioavailability, instability under physiological conditions, poor absorption from the gastrointestinal tract, and rapid metabolism [13,14,15,16], although these can be overcome with the use of nanoformulations. Nanoformulations allow precise drug delivery using nanoparticles, which can increase the biological activity of CUR and its targeting to previously inaccessible sites [17]. Research also indicates an improvement in the solubility of nanocurcumin and optimized intracellular uptake.

To date, liposomes are among the most popular nanoparticles for curcumin delivery [18]. Liposomes are spherical vesicles with a hydrophilic core encircled by phospholipid layers and are classified according to their size: small, large, and giant vesicles; number of layers: single, oligo- and multilayered; and phospholipid charge: neutral, anionic, or cationic [19].They are mainly composed of natural and/or synthetic phospholipids with amphipathic properties, a characteristic feature of which is spontaneously aggregating at the phase boundary [20]. Liposomes as drug nanocarriers provide enormous possibility for modifying physicochemical and structural properties, which features could significantly influence the drug distribution in vivo, e.g., its stability, adequate drug release, biodistribution, and cellular uptake of the liposomes [21]. Moreover, by protecting the medicinal substances through encapsulation in liposomes it is possible to prevent degradation [22].

**Figure 1:**
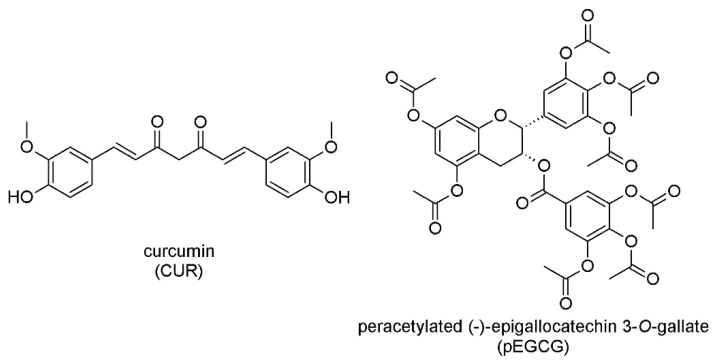
Chemical structures of curcumin ((1E,6E)-1,7-bis(4-hydroxy-3-methoxyphenyl)hepta-1,6-diene-3,5-dione; CUR) and peracetylated EGCG (3′,3″,4, 4″, 5, 5″,7-*O*-octaacetyl-(−)-epigallocatechin 3-*O*-gallate; pEGCG).

Herein, we present research on the possibility of encapsulating selected polyphenols (CUR and pEGCG) in liposomal carriers for drug delivery and combining substances to provide an advantage in anticancer potential towards urogenital cancer cell lines. Moreover, we demonstrate the methodology of preparing liposomal formulation by co-embedding two active substances, thus enhancing the time-dependent stability of compounds enclosed in those nanocarriers. The structures of the compounds used in the current study are presented in Figure 1.

## 2. Materials and Methods

### 2.1. Chemical Compounds and Reagents

Curcumin, (1E,6E)-1,7-bis(4-hydroxy-3-methoxyphenyl)hepta-1,6-diene-3,5-dione, was obtained from Fluorochem (Derbyshire, United Kingdom). 1-Palmitoyl-2-oleoyl-glycero-3-phosphocholine (POPC) and 1,2-dioleoyl-3-trimethylammonium-propane (DOTAP) were obtained from Avanti Polar Lipids (Birmingham, AL, USA).

HPLC grade acetonitrile, water, acetic acid and potassium chloride were procured from Avantor Performance Materials (Gliwice, Poland).

The peracetylated derivative of EGCG, 3′,3″,4′,4″,5,5′,5″,7-*O*-octaacetyl-(–)-epigallocatechin 3-*O*-gallate, was prepared according to a literature procedure [23].

Reagents used for in vitro experiments, such as fetal bovine serum (FBS), phosphate-buffered saline (PBS), trypsin-EDTA, L-glutamine, dimethylsulfoxide (DMSO), 3-(4,5-dimethylthiazol-2-yl)-2,5-diphenyltetrazolium bromide (MTT), were obtained from Sigma Aldrich (St. Louis, MO, USA). The DMSO for dissolving formazan crystals was obtained from Avantor Performance Materials (Gliwice, Poland). The following cell lines: 5637 (human bladder grade II carcinoma), LNCaP (human prostate carcinoma), and non-cancerous cell line MRC-5 (normal human lung fibroblast) were purchased from the American Type Culture Collection (ATCC, Manassas, VA, USA). The LNCaP and MRC-5 cell lines were maintained in DMEM medium, and 5637 in RPMI-1640 supplemented with 10% (*v*/*v*) FBS, 1% (*v*/*v*) L-glutamine (200 mM), 1% (*v*/*v*), 10 000 penicillin units, 10 mg/mL streptomycin solution. Cells were cultured at 37 °C, 5% CO_2_, and 95% humidity atmosphere.

### 2.2. Liposome Preparation

**Figure 2:**
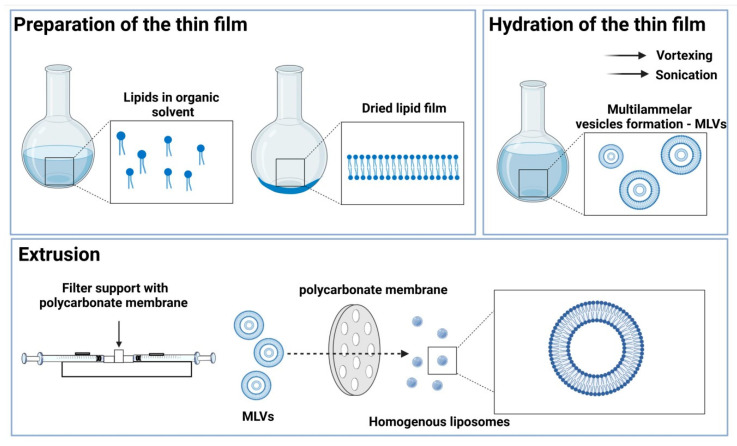
The schematic illustration of liposomes preparation protocol using the thin-film hydration followed by extrusion method. The image was prepared according to Zhang [26] and created with *BioRender.com* and Servier Medical Art (last accessed on 10 January 2022).

A modified thin-film hydration method was used to embed compounds in the liposomal formulation [24]. A schematic illustration of the preparation of liposomes via thin-film hydration is presented in Figure 2. Liposomes were prepared from 1-palmitoyl-2-oleoyl-sn-glycero-3-phosphocholine (POPC) or POPC/1,2-dioleoyl-3-trimethylammonium-propane (DOTAP) mixture (9:1 or 8:2), and either CUR, pEGCG, or their combination with a molar ratio of 1.7:10 (CUR:lipids), 1.7:10 (pEGCG:lipids), and 0.8:0.8:10 (CUR:pEGCG:lipids). The final concentrations were as follows: 614.0 μg/mL CUR, 1 324.4 μg/mL pEGCG, 7 600.8 μg/mL POPC and 307.0 + 662.2 μg/mL mixture of compounds (CUR+pEGCG). Briefly, the lipids and chemical compounds were dissolved in chloroform in the specified molar ratio, the solvent was evaporated, and the resulting film dried in a vacuum (15 min). After that, it was rehydrated using PBS, vortexed, and sonicated in an ultrasonic bath (5 min/180 W) until the uniform suspension was achieved. Next, the particle size was unified using Avanti^®^ Polar Lipids Mini Extruder (Merck KGaA, Darmstadt, Germany) with 100 nm polycarbonate membranes, according to the manufacturer’s instructions.

The encapsulation efficiency (EE) of CUR, pEGCG and their mixture was calculated according to the formula [25]:

EE = C_en_/C_in_ × 100%

where C_en_—the actual amount of the substance in the liposomes measured after their disruption using HPLC, C_in_—the initial amount of the substance used for the preparation of the liposomes.

### 2.3. Liposome Size and Zeta Potential Measurement

Liposome size was determined in PBS buffer using Nanoparticle Tracking Analysis (NTA) technique on a Nanosight LM10, equipped with a sCMOS camera and 405 nm laser, with NTA 3.2 Dev Build 3.2.16 software (Malvern Panalytical, Malvern, UK). This equipment analyzes videos captured using the instrument, giving a particle size distribution and particle count based upon tracking each particle’s Brownian motion and the size being calculated according to the Stokes-Einstein equation [27,28]. The samples of the liposomes were diluted 1:10 000 to achieve the concentration operating range of the equipment [29]. Zeta potential was measured using Zetasizer Nano ZS (Malvern Instruments, Malvern, UK). Measurements were performed in triplicate. 100 μL of liposomes were diluted with 10 mL of water.

### 2.4. HPLC Analysis

The stability studies were carried out using the HPLC method, validated for selectivity, precision, and linearity. The applied method met the accepted validation criteria. The method was developed to determine the concentration of CUR and pEGCG. The analytical apparatus comprised a 1220 Infinity LC chromatography system (Agilent Technologies, Santa Clara, CA, USA) equipped with a DAD detector, a G1315C optical unit, an autosampler, and a column oven. The gradient elution utilizes the mobile phase consisting of acetonitrile (phase A) and 1% acetic acid solution with potassium chloride (2 g/1 L, phase B) ratio 5%/95% at 0 min. Mobile phase concentration changed to 100%/0%, A/B in 30 min and returned to the starting ratio within 10 min (40 min). The stationary phase was octadecylsilyl silica gel for chromatography (Lichrospher^®^ 100 RP-18 column, 25 × 4 mm; 5 µm, Merck), stored at 25 ± 1 °C. The UV wavelength, flow rate and injection volume were 280 nm, 1.05 mL/min, and 20 μL, respectively. The retention time for CUR was 20.7 min, for pEGCG—22.0 min, and the analysis run time was 40 min. Each sample was injected in triplicate. The validation of the HPLC method concerned selectivity, precision, linearity, range, and limits of detection and quantitation was performed.

### 2.5. Biological Activity Assessment

The cytotoxic effect of the tested formulations was determined using the MTT assay [30,31], with some modifications [32]. 5637 and MRC-5 cells were seeded at a density of 15 × 10^3^ cells/well, while LNCaP cells were seeded at a density of 10 × 10^3^ cells/well in 96-well plates, and incubated overnight. The cells were treated with pEGCG and CUR (dissolved in DMSO) at the concentration of 3, 6, 12, 25, 50 and 100 µM. The DMSO was used as a control, and its concentration in the medium did not exceed 0.1%. For testing of the liposomal formulations, the tested compounds were added at a concentration of 1.2, 2.5, 5, 10, 20, and 40 µM. The combination of both compounds was tested at a concentration of 0.6, 1.2, 2.5, 5, 10, and 20 µM. The two controls were used: cell culture medium and empty liposomes (at a concentration corresponding to the higher tested dose).

The MTT assay was performed after 24 and 48 h. Briefly, the cells were washed twice with PBS, and MTT (0.59 mg/mL) was then added to each well, and incubated for 1.5 h at standard cell culture conditions. The formazan crystals were dissolved in 200 μL of DMSO, and the absorbance was measured at 570 nm with a plate reader (Biotek Instruments, Elx-800, Winooski, VT, USA). Cell viability was calculated as a percentage of the control (cell culture medium for liposomal formulation, and DMSO in cell culture medium for free form). All experiments were repeated at least three times (only pEGCG toxicity against 5637 cells was determined from two independent experiments). The IC_50_ values were determined using GraphPad 8.0 software.

### 2.6. Statistical Analysis

The statistical analysis was performed using GraphPad Prism^®^8 (GraphPad Software, Inc., La Jolla, CA, USA). One-way ANOVA with post-hoc Tukey’s test and Dunnett’s test were used to determine the significance; *p* < 0.05 was considered significant.

## 3. Results and Discussion

The rationale for including two polyphenols together in an formulation was to assess whether the individual components, which already exhibited anticancer activity, would interact to improve formulation stability, encapsulation efficiency, and reveal a synergistic effect [33,34,35,36].

Due to the instability of EGCG [12], the fully acetylated derivative of (−)-epigallocatechin 3-gallate (pEGCG) was used instead of the most commonly studied EGCG. The liposomes were prepared by a modified thin-film hydration method. The concentration of curcumin and peracetylated EGCG within the liposomes was established experimentally as both compounds reveal low biological activity. In order to achieve the desired anti-cancer effect, high concentrations in lipids were used [37], which allowed the extrusion of further resulting liposomes. Such concentration was found to be at 1 666 µM and the encapsulation efficiency was: 92.26 ± 1.19% for CUR, 54.58 ± 9.71% for pEGCG, and 91.51 ± 1.62% for CUR from the mixture (CUR+pEGCG) and 76.84 ± 0.61% of pEGCG from the mixture (CUR+pEGCG). Preparing liposomes containing higher concentrations of CUR or pEGCG resulted in too high resistance during the mechanical unification of the liposome size, which in turn caused either leaks from the equipment and subsequent loss of the formulation dispersion, or perforation of the polycarbonate membranes. The concentration of the curcumin causing disturbances in the liposomes was reported at 60 mg/L (~163 µM). This was because the lipid applied was dioleoylphosphatidylcholine, which formed smaller liposomes (65 nm) [38]. Interestingly, Wu et al. reported that the encapsulation efficiency of curcumin in the liposomes might either increase or decrease along with the increasing ratio of CUR to lipid ratio, depending on the type of phospholipids used [39].

### 3.1. Liposome Size

**Figure 3:**
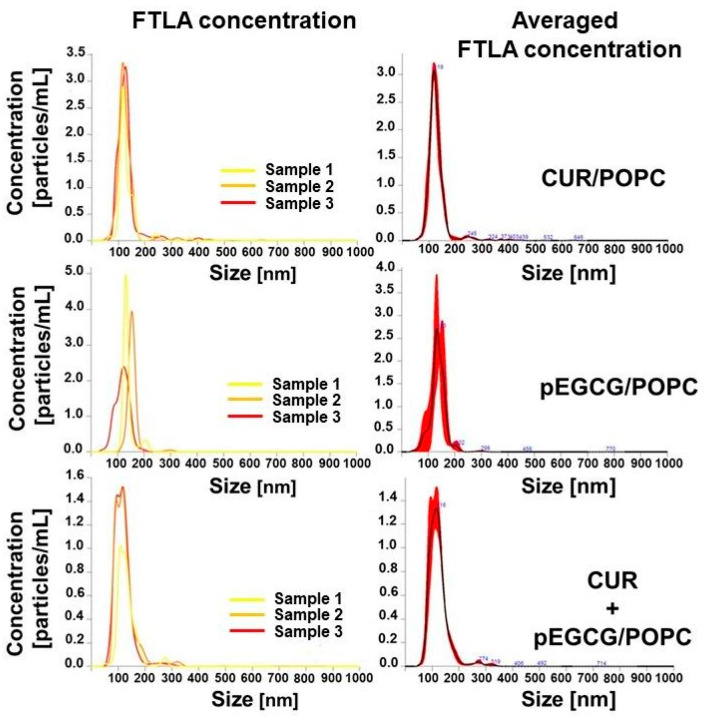
The mean hydrodynamic diameter distribution patterns of the prepared liposomes subjected to biological evaluation. FTLA—the finite track length adjustment.

The liposomes were studied in terms of their particle size to ensure the efficiency and repeatability of the obtained biological results. The size of the liposomes was measured immediately before the in vitro test, and the results are presented in Figure 3 and Table 1. The mean particle size of the prepared liposomes was about 100 nm, which is essential to increase biodistribution in potential subsequent in vivo activity studies [40,41]. In general, the particle size analyses of liposomal formulations were performed using Dynamic Light Scattering (DLS; data not shown) and NTA, while the zeta potential was determined using Electrophoretic Light Scattering (ELS) available in the Zetasizer Nano device. It should be noted that during NTA, size distribution and nanoparticle concentration are measured in real-time; thus allowing the early detection of physical instability, otherwise indeterminable if the changes in size distribution are minimal [42].

Zeta potential provides information about the surface charge properties of a nanoparticle, which can present cationic, anionic, or neutral character. According to the interpretation of the zeta potential, the values in the range of −10 to +10 mV are considered to be neutral for liposomes. Zeta potential measurements provide information about the ion concentration in the immediate vicinity of the membrane. Moreover, the magnitude of the zeta potential indicates the stability of colloidal systems [44]. In the herein presented study, zeta potential values ranged from −7.04 (pEGCG) to −4.76 (CUR) mV, which confirms the neutral character of obtained liposomes.

### 3.2. Liposomal Formulation Stability Study

#### 3.2.1. Particle Size and Particle Concentration

**Figure 4:**
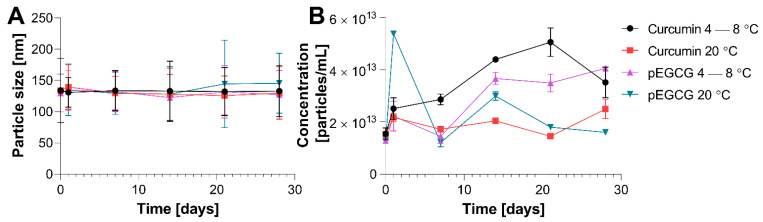
(**A**) the change of the mean liposome size over 28 days of experiment depending on the storage conditions; (**B**) the change of the mean liposome concentration over 28 days of experiment depending on the storage conditions. Error bars represent standard deviation of the mean.

The particle size and concentration of the prepared liposomes were monitored throughout 28 days of the storage experiment. The EGCG or CUR-containing liposomes were kept in the refrigerator (at 4–8 °C) or at room temperature (20 °C). The results of the study are presented in Table 2 and Figure 4.

The mean particle size of the prepared liposomes exceeded 100 nm, even though they were extruded through 100 nm carbonate filters. A possible reason for this is that upon extrusion, the liposomes could have disassembled and assembled. Another possible reason could be because of the methodology of the particle size measurement—the obtained values are for hydrodynamic diameters calculated in solution, so there might be an interaction between the solvent (water) and the liposome surface resulting in the formation of solvation (hydration) shell [45].

The increase in the liposome size over time may be due to the agglomeration of liposomes or even the merging of liposomes with each other. If multilamellar vesicles are formed, several phenomena might appear. Firstly, the layers might merge, leading to the formation of less-layered larger liposomes, which in turn may disassemble and reassemble, therefore yielding more liposomes. Another alternative is that the outer layer may detach from the liposomes and merge with another liposome thus increasing its size, and form a liposome by itself or assemble with another group of phospholipids to form a new vesicle. What can also occur is that the water trapped between the liposome layers may be expelled, leading to smaller-size liposomes [46].

The values of the polydispersity index (PDI) obtained for the prepared CUR and pEGCG loaded liposomes are less than 0.15 (except for one measurement for pEGCG on day 21 at room temperature, for which the PDI is 0.23). The FDA mentions the importance of the uniform distribution of the liposome size in its guidelines; however, there is no threshold value stated. Based on the literature data on the subject, if the PDI value does not exceed 0.3, the obtained liposome suspension is regarded as homogeneous [47,48]. Therefore, the obtained liposome mixtures are characterized by a homogeneous vesicle size distribution.

#### 3.2.2. Stability Study at Room (20 °C) and Refrigerator (4–8 °C) Temperatures

**Figure 5:**
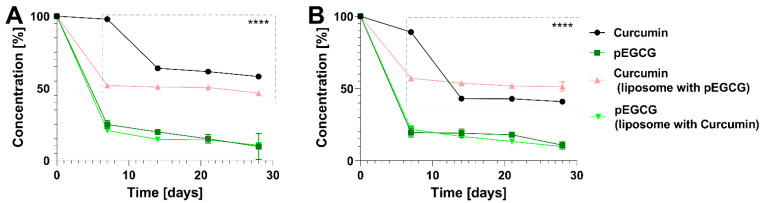
Stability study of CUR, pEGCG and their combination in liposomal nanoformulations at: (**A**) room temperature (20 °C), and (**B**) refrigerator temperature (4–8 °C). Statistical significance was assessed by Tukey’s Test (**** *p* < 0.0001). Error bars represent standard deviations of the mean.

Unexpectedly, the concentration of the curcumin in CUR/POPC showed a greater decrease when stored in the range 4–8 °C (41% of the initial value) than at the room temperature (58%). Usually, the decomposition of CUR in liposomes is faster when the temperature is higher [46]. This might be due to the increase of rigidity of lipids at a lower temperature which in turn provides easier access of CUR to water. In the case of CUR in CUR/pEGCG/POPC formulation, there was almost no difference in the concentration changes between the experiments conducted at different temperatures (Figure 5, Table 3 and Table 4). However, CUR was more stable at the refrigerator temperature in CUR+pEGCG/POPC formulation than in the CUR/POPC formulation. This might suggest that pEGCG induced a protective effect on CUR when co-embedded in the liposomes, as both exhibit highly lipophilic character and are placed in the lipid layer of the liposomes. A similar phenomenon for CUR and resveratrol has been reported [49].

It is worth noting that the greatest increase in the stability of the pEGCG is present in the prepared nanoformulation. Although the pEGCG concentration on the 28th day was as low as 10% of the initial concentration in both refrigerator and room temperature, free pEGCG is fully degraded in the culture medium at 37 °C over 120 min [50]. pEGCG decomposes rapidly over the first 7 days of storage, but the decrease is much slower over the remaining 21 days, with the concentration falling from 20 to 10%.

### 3.3. Biological Activity

The potential of phytochemicals, which might modulate numerous signaling pathways involved in cancer development and progression has been presented in several preclinical and clinical studies [51,52,53]. To date, a plethora of dietary phytochemicals, such as curcumin [54], epigallocatechin-3-*O*-gallate [55], resveratrol [56], lycopene [57], sulforaphane [58], and others, have demonstrated anticancer effects, including against prostate and bladder cancers [59,60]. Although our knowledge about cancer biology is rapidly increasing, and significant progress in cancer research has been made during recent years, drug resistance development remains one of the most critical challenges in cancer treatment [61,62]. Because of the prevailing situation drugs are often administered in combinations to overcome the aforementioned problem and increase treatment efficacy. However, there are no simple and clear solutions as to which compounds should be combined and what is more how they should be administered to maximize their effectiveness. Somers-Edgar et al. showed that a combination of EGCG and curcumin used at a lower dose might lead to synergistic anticancer activity towards triple-negative breast cancer cells [63]. Also, Eom et al. found that co-treatment of EGCG and curcumin improved anticancer effects in prostate cancer PC-3 cell line [36]. However, the literature data shows that applying these two compounds might also have an antagonistic effect in that almost all phytochemicals demonstrate low bioavailability, which may further decrease their effectiveness in clinics. Thus, tremendous efforts have been made to improve their stability, release, and membrane permeation, and protection from extensive metabolic processes. In the context of EGCG, these can be done by designing nanostructure-based drug delivery systems and specific molecular modifications [64,65]. Peracetylation is a well-known modification to protect EGCG from oxidative degradation and rapid biotransformation [65]. The advantage of pEGCG was found in both in vitro and in vivo models [66,67,68]. Lee and coworkers found that pEGCG was more effective in suppressing the growth of androgen-independent prostate cancer in nude mice than the EGCG [69]. Other researchers have described how pEGCG might serve as a novel angiogenesis inhibitor via decreasing VEGFA secretion by endometrial cancer cells through inhibiting PI3K/AKT/mTOR/HIF1α signaling pathway [70]. Numerous basic research studies support the hypothesis that a nano-scale delivery system might improve EGCG absorption. To date, several carrier systems, including lipid-based, polymer-based, carbohydrate-based, protein-based, and metal-based nanoparticles, have been used to promote EGCG stability and absorption [71]. However, there is limited knowledge about the encapsulation of pEGCG. Thus, in the presented study, we used pEGCG instead of the commonly used EGCG [70]. Curcumin is a well-known compound that possesses pleiotropic activity, including anticancer activity. However, curcumin has not yet been approved as a therapeutic agent due to its low solubility, bioavailability, and rapid metabolism [72,73]. Like EGCG, several possibilities for overcoming drawbacks related to the insufficient absorption of curcumin have been proposed, including its structural modification and application with a proper delivery system. It was evidenced that encapsulation of curcumin in liposomes might improve its efficacy [74]. As Huang and co-workers nicely presented, curcumin can locate in the hydrophobic core, therefore rigidifying the entire lipid bilayers and, in this way, enhancing the stability of both liposomes and encapsulated compound [49]. To date, different liposomal formulations have been used to deliver curcumin. It should be highlighted the POPC-liposomes containing both curcumin and pEGCG as active compounds were presented in the herein study for the first time.

**Figure 6:**
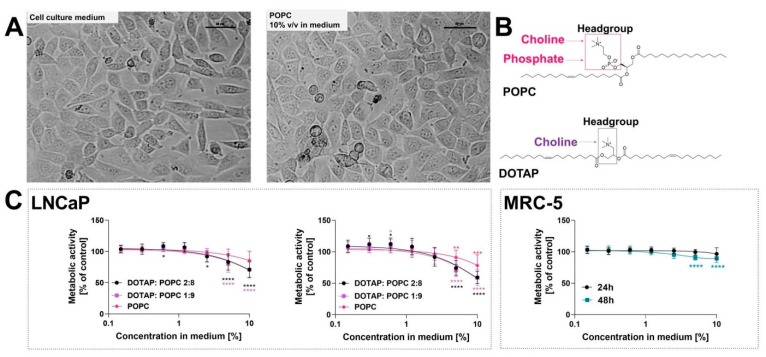
The cytotoxicity of the tested liposomal formulation. Panel (**A**) presents the cell morphology after incubation with POPC liposomes and cell culture medium after 48 h incubation time (representative images). The images were taken with a DS-SMc digital camera attached to a Nikon Eclipse TS100 microscope. Scale bar corresponds to 50 µm; panel (**B**) presents the chemical structure of 1-palmitoyl-2-oleoyl-sn-glycero-3-phosphocholine (POPC) and 1,2-dioleoyl-3-trimethylammonium-propane (DOTAP); panel (**C**) presents the effect of the tested empty liposomal formulations on cell viability. The DOTAP:POPC 1:9, DOTAP:POPC 2:8, and POPC liposomes were added to a cell culture medium at a concentration of 0.15–10% *v*/*v* for 24 and 48 h. The cell viability was measured by MTT assay. A cell culture medium was used as a control. The selected formulation was tested on a non-cancerous human lung fibroblast MRC-5 cell line. Data are expressed as the mean ± SD from five experiments (POPC, LNCaP), four experiments (DOTAP:POPC, LNCaP), and three experiments (for MRC-5). Statistical significance between groups was assessed by Dunnett’s Multiple Comparison Test (* *p* < 0.05; ** *p* < 0.01, *** *p* < 0.001; **** *p* < 0.0001).

Firstly, three liposomal formulations were tested to select the non-toxic concentration range. These experiments were performed using the human androgen-dependent prostate LNCaP cells. Following the study, DOTAP:POPC-based liposomes decreased the cell viability, regardless of the ratio of POPC to DOTAP (Figure 6). Thus, only the POPC-based formulation was used for further studies. Moreover, this formulation was also tested using a non-cancerous lung fibroblast MRC-5 cell line. The POPC at a concentration of 10% decreased MRC-5 viability to 96 and 89.71% after incubation lasting 24 and 48 h, respectively. Based on these data, the POPC concentration should not exceed 5%.

To determine the cytotoxic activity of tested compounds in their free form, the 5637, LNCaP, and MRC-5 cells were treated with CUR and pEGCG dissolved in DMSO (Table 5). The most sensitive to curcumin was the 5637 cell line with IC_50_ values of 17.95 ± 6.68 µM and 11.25 ± 2.47 µM after incubation lasting 24 h, and 48 h, respectively. Our findings align with data reported by Konstantinov and co-workers, where the curcumin IC_50_ was 14.28 μM for 5637 cells [75]. Also, Hauser et al. showed the cytotoxic activity of curcumin towards a panel of urothelial bladder cancer cell lines after treatment lasting 48 h (IC_50_ value of 10.5 ± 1 µM for 5637 cells) [76]. In our work, the IC_50_ values for LNCaP cells were 30.61 ± 6.95 µM and 19.66 ± 3.78 μM after 24 and 48 h of curcumin treatment, respectively. Choi and co-workers also observed similar activity against LNCaP cells. The antiproliferative effect of curcumin was measured using the MTS assay, and the IC_50_ of curcumin after 24 and 48 h treatment was 25.0 and 18.4 μM, respectively [77]. Also, Eslami et al. showed that the IC_50_ for curcumin using the MTT assay was 25.01 and 18.66 μM for 24 and 48 h, respectively [78]. In our study, the highest IC_50_ was calculated for normal human lung fibroblast MRC-5 (38.73 ± 3.39 µM and 25.81 ± 1.61 μM) for 24 and 48 h, respectively). Dhima et al. focused on curcumin’s ability to sensitize leiomyosarcoma (LMS) cells to cisplatin and also determined the cytotoxicity against MRC-5 cells with the IC_50_ value of 47.2 ± 5.1 µM after 48 h of treatment [79]. On the other hand, Muthoosamy et al. did not observe the cytotoxic effect of curcumin in MRC-5 cells (the IC_50_ > 200 µg/mL) [80]. The literature data shows that cancer cells are more sensitive to curcumin than normal cells. However, curcumin may also affect normal cells depending on the cell types. It was found that curcumin at a concentration of 10 µM might inhibit cellular proliferation through G2/M cell cycle phase arrest in human dermal fibroblasts (HDFs) [81].

The pEGCG exerted less cytotoxic activity against all tested cells than curcumin. Interestingly, the pECGC expressed higher activity against LNCaP cells, while the 5637 cells were less sensitive to pEGCG. Shenouda et al. reported that the IC_50_ of EGCG on LNCaP cells was 100 μM [82], while Luo et al. showed that EGCG inhibited the growth of 5637 cells with an IC_50_ value of 69.5 μM [83]. However, there is no research indicating the cytotoxicity of peracetylated EGCG against LNCaP and 5637 cell lines.

**Figure 7:**
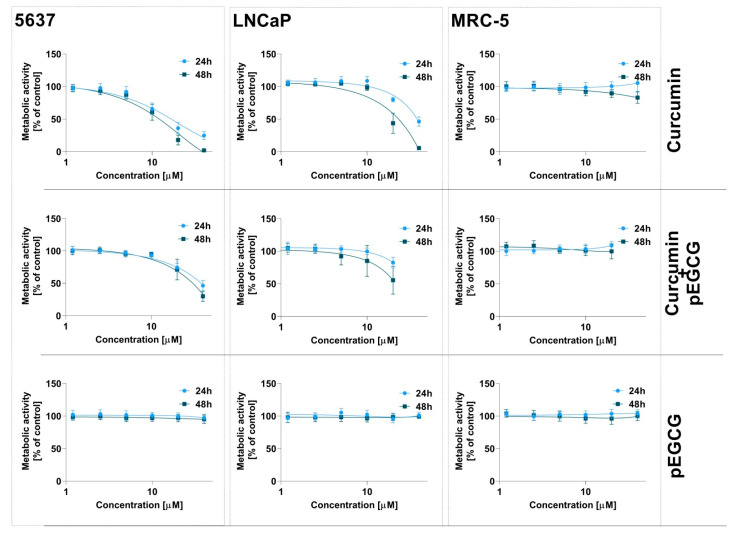
The cytotoxic activity of liposomal formulation containing curcumin, pEGCG, and their combination against 5637, LNCaP, and MRC-5 cells. Data are expressed as the mean ± SD from three independent experiments.

The IC_50_ values of the liposomal formulation of tested compounds and their combination are presented in Table 6, and the dose-response curves are presented in Figure 7. The IC_50_ values for curcumin and curcumin encapsulated in POPC liposomes are comparable for 5637 cells. For the free form, the IC_50_ values were 17.95 ± 6.68 µM and 11.25 ± 2.47 µM for 24 and 48 h, respectively; while for the liposomal formulation, IC_50_ reached the values of 17.12 ± 4.09 µM and 12.27 ± 2.91 µM for 24 and 48 h incubation time, respectively. LNCaP cells also responded similarly to curcumin treatment. At the doses used for this experiment, pEGCG did not significantly affect the viability of either of the cell lines. These results clearly show that it is CUR, which is responsible for the cytotoxic effects within this combination of ingredients. For 5637 and LNCaP cells, the curcumin showed comparable suppression of cell viability for liposomal formulation and the free form in DMSO. Interestingly, the cytotoxic activity of curcumin against normal fibroblast was lower in liposomal formulation compared to curcumin dissolved in DMSO. To date, the curcumin-loaded POPC-based liposomes were studied towards the protective activity against dental pulp stem cells (hDPSCs) [84]. The authors found that curcumin in POPC at the concentration of 20 µM did not affect cell viability. Moreover, liposomal formulation increased hDPSCs’ proliferation and inhibited inflammatory cytokines secretion by regulating NFkB/ERK and pERK signaling cascades [84]. The POPC-based liposomes were also used to design the theranostic system that combined curcumin and bis(2,4,6-trichlorphenyl)oxalate (TCPO) to detect oxidative stress in cancer cells [85]. Curcumin acts as a fluorochrome in this system, while TCPO is an inducer for peroxyoxalate chemiluminescence (PO-CL) reaction [85]. In the proposed mechanism for PO-CL reaction, an oxalic acid derivative (TCPO) reacts with hydrogen peroxide to generate intermediate 1,2-dioxetanedione, which does not emit light but transfers its energy to a fluorescent molecule (curcumin) emitting light after relaxation to the ground state [85]. However, both curcumin and TCPO are hydrophobic and degrade in an aqueous environment; the POPC-based liposomal formulation was used to deliver this cargo into cells. Moreover, the authors reported that the interaction of curcumin with the POPC liposomes might stabilize the structure and minimize curcumin degradation [85]. However, there is no evidence of cytotoxicity of curcumin–POPC liposomes against bladder and prostate cancer cells.

The presented results confirm the possibility of using POPC liposomes instead of DMSO solutions in vitro. These results also suggest that the tested formulation might be considered for in vivo experiments. However, further in vivo studies should be performed to verify the lack of toxicity of the empty liposomal formulation. Scientific research demonstrates the negative impact of DMSO on biological assays. Verheijen et al. demonstrated the ability of DMSO to induce changes in cellular processes, which may significantly influence the formulated conclusions, e.g., regarding drug toxicity [86]. Galvao and co-workers reported that the final concentration of DMSO, even used at a low concentration—0.1% (*v*/*v*)—is toxic in vivo and leads to significant retinal apoptosis [87]. Thus, other formulations, e.g., liposomes, are more favorable than DMSO as a drug solvent. The authors also recommended that the percentage of DMSO used to dissolve drugs be kept to a minimum (1% *v/v* solutions for injections) and highlighted the need to include an additional untreated control group to verify the potential solvent toxic effects [87]. Therefore, the use of liposomes fits perfectly with the 3Rs rule (reducement, refinement, replacement) by reducing the number of animals needed for study and avoiding potential side effects. To obtain the desirable results it is important to consider the injection of pEGCG or curcumin, which would have to be used at relatively high concentrations.

**Figure 8:**
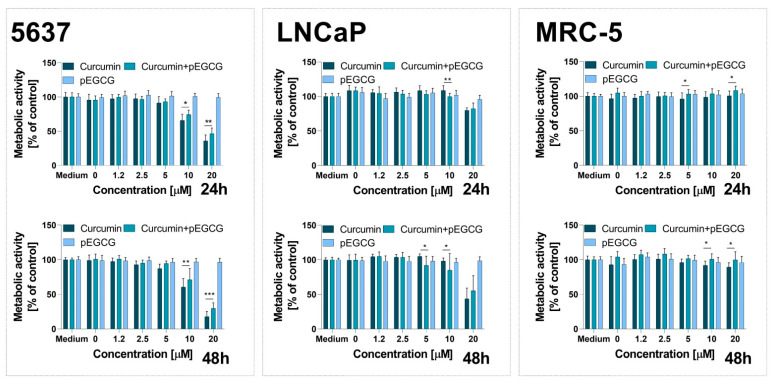
The comparison of curcumin, pEGCG, and combination of both compounds in liposomes against 5637, LNCaP, and MRC-5 cells. Data are expressed as the mean ± SD from three experiments. Statistical significance between groups was assessed by Tukey’s Comparison Test (* *p* < 0.05; ** *p* < 0.01, *** *p* < 0.001).

Phytochemicals might interact with each other, promoting the effectiveness of the compounds via synergistic or additive effects. However, different bioactive compounds might also block or reduce their activity (antagonistic effect) [88]. The observed combinatorial effect of CUR and pEGCG was slightly antagonistic rather than synergistic (Figure 8). The combination of CUR with pEGCG at the tested concentration range led to the decreased activity of curcumin in 5637 and LNCaP cells. As was noted by Ghosh et al., administration of curcumin simultaneously with EGCG is associated with antagonistic activity towards primary chronic lymphocytic leukemia (CLL) B cells [89]. In contrast, sequential administration of these compounds increases cell death via apoptosis pathway, compared to the treatment with the individual agent alone [89]. Thus, it appears that pEGCG might exert a similar effect as EGCG when it comes to decreasing CUR activity. Admittedly, our study is not free from several limitations, including only one tested molar ratio of CUR and pEGCG and only simultaneous administration of both compounds. It is well-known that after administration, firstly, each nanoparticle will be exposed to a complex biological fluid, such as serum, that is rich in various proteins. Thus, the liposome surface is coated mainly by proteins to form a biomolecular corona (BMC) in the living system [90], and this phenomenon might affect liposome stability and size [91,92,93]. Thus, considering this critical aspect of the liposomal-based drug delivery, further studies focusing on the behavior of tested formulations in cell culture medium must be performed to better predict their potential use in in vivo models. However, our findings present important knowledge about the potential interaction of CUR and pEGCG used at relatively low concentrations. Furthermore, our results showed that curcumin encapsulated in POPC exerted a lower cytotoxic effect against non-cancerous cells.

## 4. Conclusions

The presented studies proved the possibility of encapsulating two different polyphenols (CUR and pEGCG) in liposomes (POPC) at two temperature levels, i.e., at room temperature (20 °C) and in a refrigerator (4–8 °C). Such a combination allowed to increase the stability of CUR in CUR + pEGCG/POPC formulation to a more significant extent than in CUR/POPC formulation at refrigerator temperature (51 and 41% of the initial concentration, respectively). In addition, the closed environment provided by liposomes, increases stability of pEGCGs and does not lead to complete degradation, resulting in 10–11% of the initial concentration of pEGCG at the end of the studied period. Notably, the maximum concentration of CUR in POPC liposomes has been designated at 1 666 µM, limiting the possible toxic effects of lipids on cells. What is more, the cytotoxic effect of liposome-encapsulated CUR on bladder cancer cells (5637 cell line) was demonstrated for the first time with IC_50_ calculated at 19.50 ± 3.23 µM and 15.33 ± 2.03 µM after 24 and 48 h, respectively. Moreover, the presented results confirm the possibility of using POPC liposomes instead of DMSO solutions in the in vitro tests, which has great significance when translating the in vitro to in vivo studies. The presented research provides new possibilities for applying selected polyphenols to urogenital neoplasms, including the use of the potential provided by the encapsulation in liposomes to targeted therapy. This methodology could be a promising tool to increase the concentration of CUR and pEGCG through their precise delivery into cancer cells.

## Figures and Tables

**Table 1:** The size of the liposomes and their concentration as prepared immediately before the biological activity study.

Compound	Particle Size (±SD) [nm]	PDI ^a^	Concentration (±SD) [Particles/mL]
CUR	129.9 ± 45.0	0.120	1.48 × 10^13^ ± 1.87 × 10^12^
pEGCG	136.8 ± 33.5	0.060	1.39 × 10^13^ ± 8.47 × 10^11^
CUR+pEGCG	123.3 ± 41.8	0.115	8.89 × 10^12^ ± 1.29 × 10^12^

^a^—polydispersity index calculated according to the formula PDI = (SD/particle size)^2^ [43].

**Table 2:** The size of the liposomes and their concentration depending on the storage conditions and embedded polyphenol.

**Temperature**	**Curcumin**			
	**Storage Time [Days]**	**Particle Size (±SD) [nm]**	**PDI ^a^**	**Concentration (±SD) [Particles/mL]**
4–8 °C	0	133.8 ± 51.5	0.148	1.54 × 10^13^ ± 2.31 × 10^12^
	1	130.9 ± 23.6	0.033	2.51 × 10^13^ ± 4.11 × 10^12^
	7	133.6 ± 32.2	0.058	2.86 × 10^13^ ± 2.07 × 10^12^
	14	133.0 ± 48.0	0.130	4.39 × 10^13^ ± 1.04 × 10^12^
	21	132.1 ± 38.4	0.085	5.05 × 10^13^ ± 5.45 × 10^12^
	28	133.1 ± 41.1	0.095	3.51 × 10^13^ ± 5.87 × 10^12^
20 °C	1	139.4 ± 35.5	0.065	2.17 × 10^13^ ± 1.63 × 10^12^
	7	130.1 ± 26.5	0.041	1.72 × 10^13^ ± 2.44 × 10^11^
	14	128.0 ± 41.6	0.106	2.04 × 10^13^ ± 8.49 × 10^11^
	21	125.8 ± 31.3	0.062	1.45 × 10^13^ ± 4.75 × 10^11^
	28	129.7 ± 42.5	0.107	2.49 × 10^13^ ± 3.66 × 10^12^
**Temperature**	**pEGCG**			
	**Storage Time [Days]**	**Particle Size (±SD) [nm]**	**PDI ^a^**	**Concentration (±SD) [Particles/mL]**
4–8 °C	0	129.8 ± 30.3	0.054	1.28 × 10^13^ ± 8.67 × 10^11^
	1	134.0 ± 31.6	0.056	2.22 × 10^13^ ± 5.67 × 10^12^
	7	132.2 ± 32.0	0.059	1.45 × 10^13^ ± 2.73 × 10^12^
	14	122.7 ± 36.9	0.090	3.66 × 10^13^ ± 2.39 × 10^12^
	21	131.7 ± 41.9	0.101	3.49 × 10^13^ ± 3.37 × 10^12^
	28	127.2 ± 38.6	0.092	4.05 × 10^13^ ± 9.78 × 10^11^
20 °C	1	135.1 ± 41.2	0.093	5.37 × 10^13^ ± 5.03 × 10^11^
	7	129.5 ± 33.7	0.068	1.21 × 10^13^ ± 1.60 × 10^12^
	14	128.2 ± 44.2	0.119	2.99 × 10^13^ ± 1.62 × 10^12^
	21	144.4 ± 70.1	0.236	1.80 × 10^13^ ± 4.22 × 10^11^
	28	145.6 ± 48.0	0.109	1.60 × 10^13^ ± 1.04 × 10^12^

^a^—polydispersity index calculated according to the formula PDI = (SD/particle size)^2^ [43].

**Table 3:** Changes in the concentration of CUR and pEGCG in liposomal nanoformulations in the stability study in room temperature (20 °C).

Time [Days]	CUR C/C_0_	SD	pEGCG C/C_0_	SD	CUR C/C_0_ (CUR+pEGCG)	SD	pEGCG C/C_0_ (CUR+pEGCG)	SD
0	100.00%	0.18%	100.00%	0.84%	100.00%	0.61%	100.00%	1.58%
7	97.93%	0.61%	24.98%	2.81%	51.96%	0.62%	20.76%	0.38%
14	63.92%	1.35%	19.82%	0.26%	50.95%	0.50%	14.64%	0.46%
21	61.64%	0.05%	15.03%	2.89%	50.65%	1.37%	14.30%	0.00%
28	58.21%	0.90%	9.67%	9.01%	46.67%	0.72%	10.65%	0.47%

C/C_0_ is the ratio between the concentration tested at a certain time point to the initial concentration at the beginning of the experiment. SD—standard deviation.

**Table 4:** Changes in the concentration of CUR and pEGCG in liposomal nanoformulations in the stability study at refrigerator temperature (4–8 °C).

Time [Days]	CUR C/C_0_	SD	pEGCG C/C_0_	SD	CUR C/C_0_ (CUR+pEGCG)	SD	pEGCG C/C_0_ (CUR+pEGCG)	SD
0	100.00%	0.18%	100.00%	0.84%	100.00%	0.61%	100.00%	1.58%
7	89.16%	1.48%	19.58%	3.09%	57.15%	0.18%	21.81%	2.52%
14	43.00%	0.09%	19.07%	2.93%	53.67%	0.03%	16.82%	0.10%
21	42.84%	1.09%	17.98%	0.86%	51.81%	0.31%	13.27%	1.13%
28	40.94%	1.06%	10.77%	2.72%	51.27%	3.38%	9.87%	0.34%

C/C_0_ is the ratio between the concentration tested at a certain timepoint to the initial concentration at the beginning of the experiment. SD—standard deviation.

**Table 5:** The IC_50_ values of curcumin, pEGCG, and their combinations dissolved in DMSO. Data are expressed as the mean ± SD from at least three independent experiments; only pEGCG for 5637 cell line presents mean from two independent experiments.

	IC_50_ [µM]			
Cell Line	Curcumin		pEGCG	
	24 h	48 h	24 h	48 h
5637	17.95 ± 6.68	11.25 ± 2.47	84.08 ± 1.09	76.73 ± 0.24
LNCaP	30.61 ± 6.95	19.66 ± 3.78	62.45 ± 12.10	60.98 ± 9.25
MRC-5	38.73 ± 3.39	25.81 ± 1.61	73.82 ± 6.23	65.55 ± 5.92

**Table 6:** The IC_50_ values of curcumin, pEGCG, and their combination in liposomes. Data are expressed as the mean ± SD from three independent experiments.

Cell Line	Curcumin		pEGCG		Curcumin+pEGCG	
	24 h	48 h	24 h	48 h	24 h	48 h
5637	17.12 ± 4.09	12.27 ± 2.91	>40	>40	19.50 ± 3.23	15.33 ± 2.03
LNCaP	38.96 ± 2.90	22.06 ± 3.14	>40	>40	>40	>40
MRC-5	>40	>40	>40	>40	>40	>40

## Data Availability

All the data obtained in this study is presented in the article.
